# Implication of *KRT16*, *FAM129A* and *HKDC1* genes as ATF4 regulated components of the integrated stress response

**DOI:** 10.1371/journal.pone.0191107

**Published:** 2018-02-08

**Authors:** Alexandra G. Evstafieva, Irina E. Kovaleva, Maria S. Shoshinova, Andrei V. Budanov, Peter M. Chumakov

**Affiliations:** 1 Belozersky Institute of Physical and Chemical Biology, Lomonosov Moscow State University, Moscow, Russia; 2 Department of Bioengineering and Bioinformatics, Lomonosov Moscow State University, Moscow, Russia; 3 Engelhardt Institute of Molecular Biology, Russian Academy of Sciences, Moscow, Russia; 4 School of Biochemistry and Immunology, Trinity Biomedical Sciences Institute, Trinity College, Dublin 2, Dublin, Ireland; 5 Chumakov Institute of Poliomyelitis and Viral Encephalitides, Federal Scientific Center for Research and Development of Immune-Biology Products, Russian Academy of Sciences, Moscow, Russia; Virginia Commonwealth University, UNITED STATES

## Abstract

The ATF4 transcription factor is a key regulator of the adaptive integrated stress response (ISR) induced by various stresses and pathologies. Identification of novel transcription targets of ATF4 during ISR would contribute to the understanding of adaptive networks and help to identify novel therapeutic targets. We were previously searching for genes that display an inverse regulation mode by the transcription factors ATF4 and p53 in response to mitochondrial respiration chain complex III inhibition. Among the selected candidates the human genes for cytokeratine 16 (*KRT16*), anti-apoptotic protein Niban (*FAM129A*) and hexokinase *HKDC1* have been found highly responsive to ATF4 overexpression. Here we explored potential roles of the induction of *KRT16*, *FAM129A* and *HKDC1* genes in ISR. As verified by RT-qPCR, a dysfunction of mitochondrial respiration chain and ER stress resulted in a partially ATF4-dependent stimulation of *KRT16*, *FAM129A* and *HKDC1* expression in the HCT116 colon carcinoma cell line. ISRIB, a specific inhibitor of ISR, was able to downregulate the ER stress-induced levels of *KRT16*, *FAM129A* and *HKDC1* transcripts. An inhibition of ATF4 by RNAi attenuated the induction of *KRT16*, *FAM129A* and *HKDC1* mRNAs in response to ER stress or to a dysfunctional mitochondrial respiration. The similar induction of the three genes was observed in another tumor-derived cervical carcinoma cell line HeLa. However, in HaCaT and HEK293T cells that display transformed phenotypes, but do not originate from patient-derived tumors, the ER stress-inducing treatments resulted in an upregulation of *FAM129A* and *HKDC1*, but not *KRT16* transcripts, By a luciferase reporter approach we identified a highly active ATF4-responsive element within the upstream region of the *KRT16* gene. The results suggest a conditional regulation of *KRT16* gene by ATF4 that may be inhibited in normal cells, but engaged during cancer progression. Potential roles of *KRT16*, *FAM129A* and *HKDC1* genes upregulation in adaptive stress responses and pathologies are discussed.

## Introduction

One of the major adaptive signaling pathways of eukaryotic cells acts through a regulation of eukaryotic translation initiation factor 2 alpha (eIF2α). Various stressful signals converge to at least four protein kinases that phosphorylate eIF2 α leading to a global reduction in translation initiation (reviewed in [1]). Besides, the phosphorylation of eIF2 α is associated with switching to translation of selected transcripts that contain upstream open reading frames (uORF) [1, 2]. One of such preferentially translated transcripts encodes Activating Transcription Factor 4 (ATF4) that participates in the reprogramming gene expression to endure stresses [3]. Because eIF2 α phosphorylation can direct translational control in response to diverse environmental stresses, the eIF2 α /ATF4 pathway is often referred to as the integrated stress response (ISR) [3].

ATF4 is basic leucine zipper transcription factor that binds to the C/EBP-ATF response elements (CARE) composed of a half-site for the C/EBP and a half-site for a member of the ATF family transcription factors [4]. Products of the ATF4-induced genes modulate a wide spectrum of cellular processes to endure diverse stresses. Activities of the ATF4 affect amino acid biosynthesis and transport [2, 3], upregulate protein synthesis [5] and alleviate outcomes of oxidative stress [2]. The ATF4-mediated ISR draw particular attention for its role in many pathologies, including cardiac [6], neurodegenerative (Alzheimer [7], Parkinson [8]), pulmonary [9], renal [10], and several other. Various functions of ATF4 promote metabolic homeostasis and cancer cell survival in hypoxic- and nutrient-deprived regions, in particular by affecting amino acid uptake and biosynthesis, autophagy, redox balance and angiogenesis [11]. Because the ATF4 expression is increased in many tumors the ISR signaling pathway is being considered as an attractive target for anti-cancer therapy [11]. An upregulation of the ATF4 protein in response to different stress conditions usually occurs at both translation and transcription levels and is associated with the induction of ATF4 mRNA [12]. Further studies on identification of novel transcription targets of ATF4 could contribute to the development of new therapeutic strategies.

Here we describe the identification of *KRT16*, *FAM129A* and *HKDC1* genes as novel targets for the transcription regulation by ATF4. *KRT16* gene encodes a cytokeratin protein associated with hyperproliferation of keratinocytes [13]. *HKDC1* is a human hexokinase gene that is overexpressed in tumor tissues and is considered to be a promising target for a treatment of lung cancer [14]. *FAM129A* gene encodes a Niban protein that is overexpressed in many types of cancer and supposedly protects cells from apoptosis [15, 16]. We show that the ATF4-mediated induction of *KRT16*, *FAM129A* and *HKDC1* mRNAs occurs in response to endoplasmic reticulum (ER) stress and after an inhibition of mitochondrial respiration chain. We demonstrate some peculiarities in the responses that are potentially related to the process of selection of malignant cells and cancer progression.

## Results

### Identification of new potential ATF4-regulated genes

Recently we have described an algorithm for searching novel ATF4-regulated genes induced in response to an inhibition of mitochondrial electron transfer chain (ETC) [17]. The algorithm is based on the experimental evidence that in the absence of the p53 response a dysfunction of mitochondrial respiration leads to increased expression of the *ATF4* gene and of some known ATF4- regulated genes, while a simultaneous activation of p53 suppresses the ATF4 mediated transcription [18]. As the result, a transient inhibition of respiratory complex III is associated with an upregulation of ATF4 and ISR, though a more sustained dysfunction results in an activation of the p53 response due to the impaired pyrimidine biosynthesis by the ETC-coupled dihydroorotate dehydrogenase and prevents ATF4 upregulation [18–20]. The p53 response can be prevented by uridine supplementation leading to an uninterrupted activation of the ATF4-dependent ISR during the sustained inhibition of complex III [18]. A set of transcripts that are most heavily upregulated by the complex III inhibition in the absence of p53 activation were selected on the basis of transcriptome sequencing as products of putative ATF4 stimulated genes and the effect was verified by RT-qPCR in samples overexpressing ATF4 [17]. In the previous study we have revealed transcripts from *ADM2*, *KRT16*, *FAM129A* and *HKDC1* genes as most responsive to the ATF4 overexpression [17]. In ATF4 overexpressing HeLa cells levels of *ADM2*, *FAM129A*, *KRT16* and *HKDC1* mRNAs were increased 11, 9.5, 5.2 and 3.4 fold, respectively. The role of *ADM2* has been previously reported [17]. In the present study we address a significance of *KRT16*, *FAM129A* and *HKDC1* genes in ISR and discuss their potential roles in pathologies.

### Induction of mRNA from *KRT16*, *FAM129A* and *HKDC1* genes in response to different stresses

**Fig 1:**
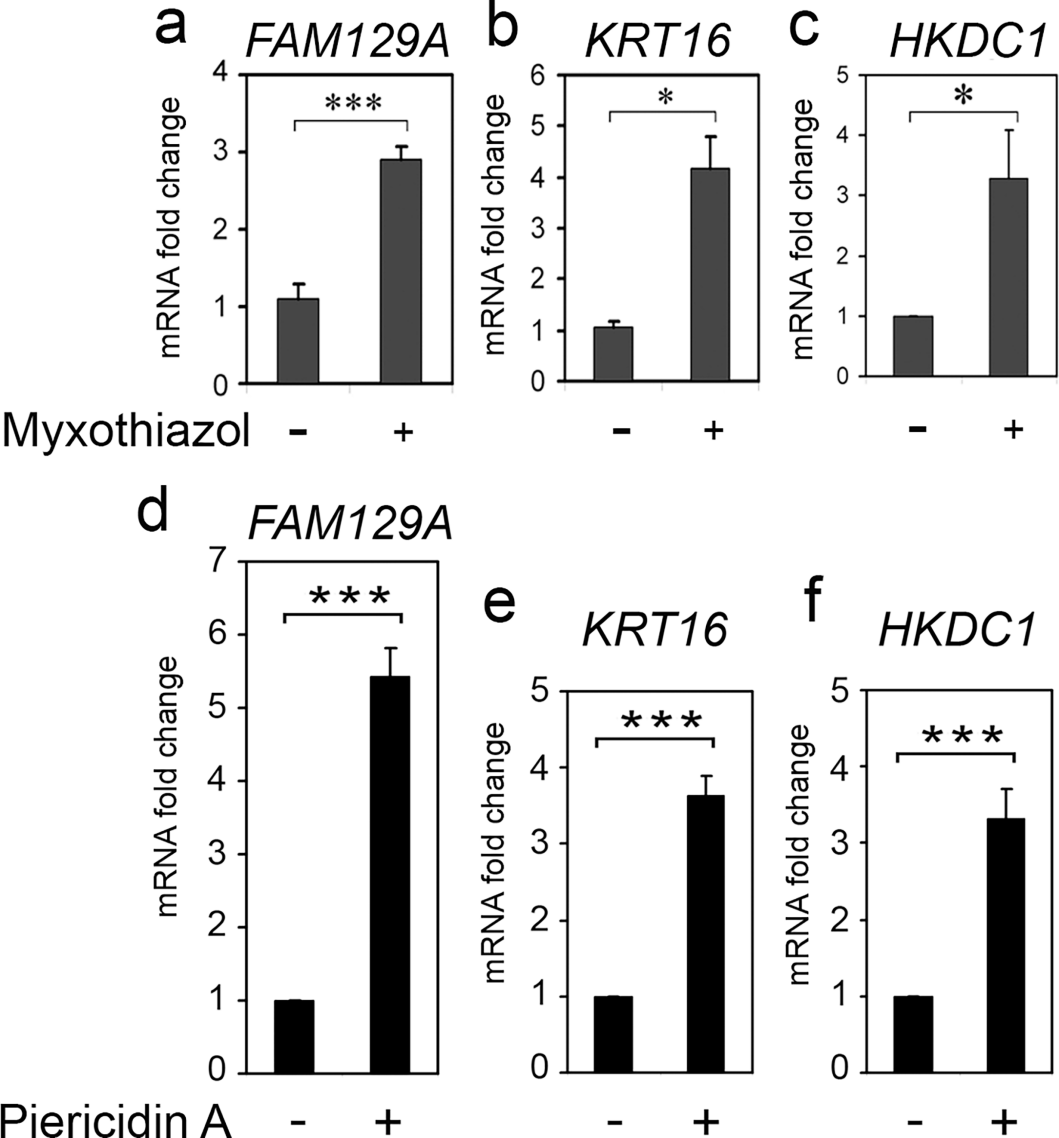
Induction of KRT16, FAM129A and HKDC1 transcripts by the inhibition of mitochondrial respiratory chain complexes I or III. Fold changes of KRT16, FAM129A and HKDC1 transcripts in HCT116 cells treated with complex III inhibitor Myxothiazol for 5 h (a, b, c) or with complex I inhibitor Piericidin A for 13 h (d, e, f). The data was obtained by RT-qPCR and processed as described in Materials and Methods.

The induction of KRT16, FAM129A and HKDC1 transcripts in HCT116 colon carcinoma cells was tested by RT-qPCR after the treatment with mitochondrial respiratory chain complex I inhibitor Piericidin A or complex III inhibitor Myxothiazol under the previously optimized conditions that result in the induction of ATF4 but do not activate p53 [18]. We observed a substantial upregulation of mRNAs from the genes (Fig 1A–1F) indicating that the inhibition of mitochondrial respiratory chain induces a coordinated stimulation of both ATF4 and transcripts from the *KRT16*, *FAM129A* and *HKDC1* genes.

**Fig 2:**
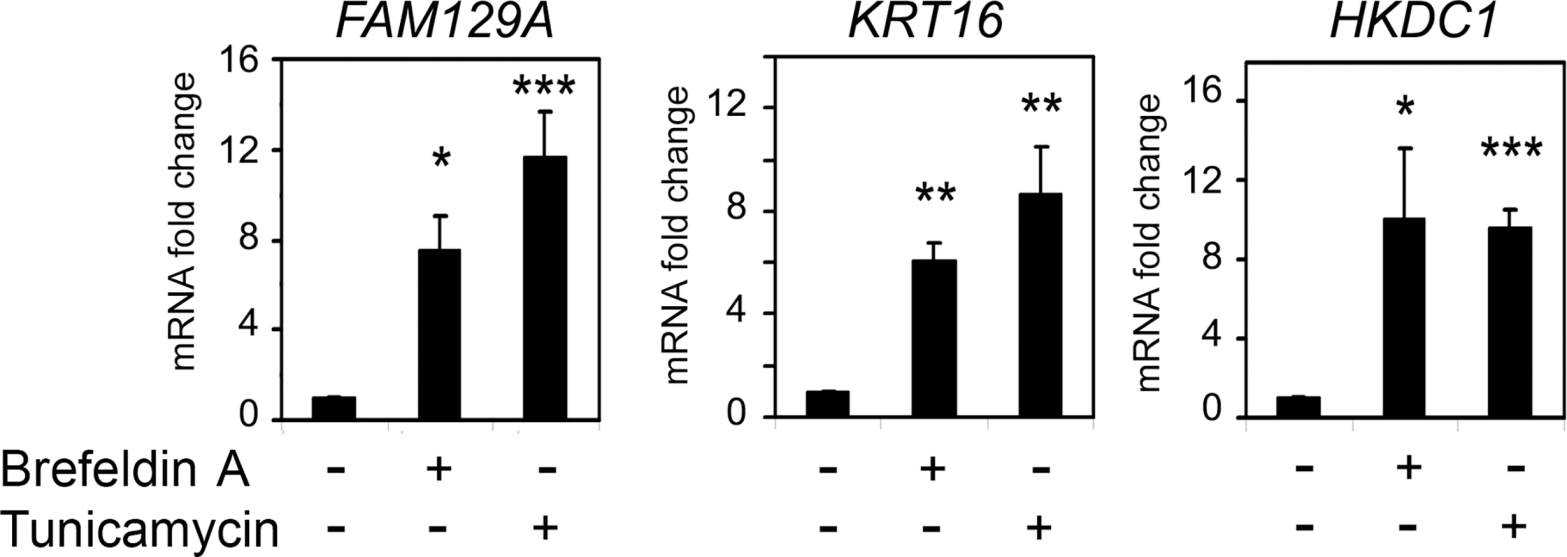
Induction of KRT16, FAM129A and HKDC1 transcripts by the UPR to ER stress. Fold changes of KRT16, FAM129A and HKDC1 transcripts in HCT116 cells treated with Brefeldin A or Tunicamycin for 14 h. The data was obtained by RT-qPCR and processed as described in Materials and Methods.

Accumulating evidences suggest that ATF4 may play a leading role in ER stress. The unfolded protein response (UPR), which is characteristic to ER stress, is induced by denatured proteins within the ER and initiates generalized signaling cascades [21, 22]. The UPR fires signaling processes that include PERK/eIF2α/ATF4 pathway [21]. During the ER stress GRP78, the master regulator of UPR, binds to unfolded proteins and releases UPR mediator, the PKR-like endoplasmic reticulum kinase (PERK), which forms dimers and phosphorylates eIF2α. The phosphorylated eIF2α activates ATF4 to facilitate transcription of ATF4-responsive genes [22]. To find whether *KRT16*, *FAM129A* and *HKDC1* genes can also respond to ER stress we tested levels of corresponding transcripts after inducing UPR by Tunicamycin and Brefeldin A, the treatments that were previously found optimal for the induction of ATF4 transcripts [17]. A substantial increase in KRT16, FAM129A and HKDC1 transcripts in HCT116 cells was observed after the treatment (Fig 2).

The results were in agreement with the suggestion that the genes under the study may indeed be regulated by ATF4 and therefore belong to ISR. To test this possibility we treated the cells with ISRIB, a specific small molecule inhibitor of integrated stress response [23]. The ISRIB was previously shown to reverse the effects of eIF2α phosphorylation on translation; in particular, ISRIB recovers the total protein biosynthesis under stress conditions, while suppressing translation of some uORF-containing transcripts, including the ATF4 mRNA [23–25].

**Fig 3:**
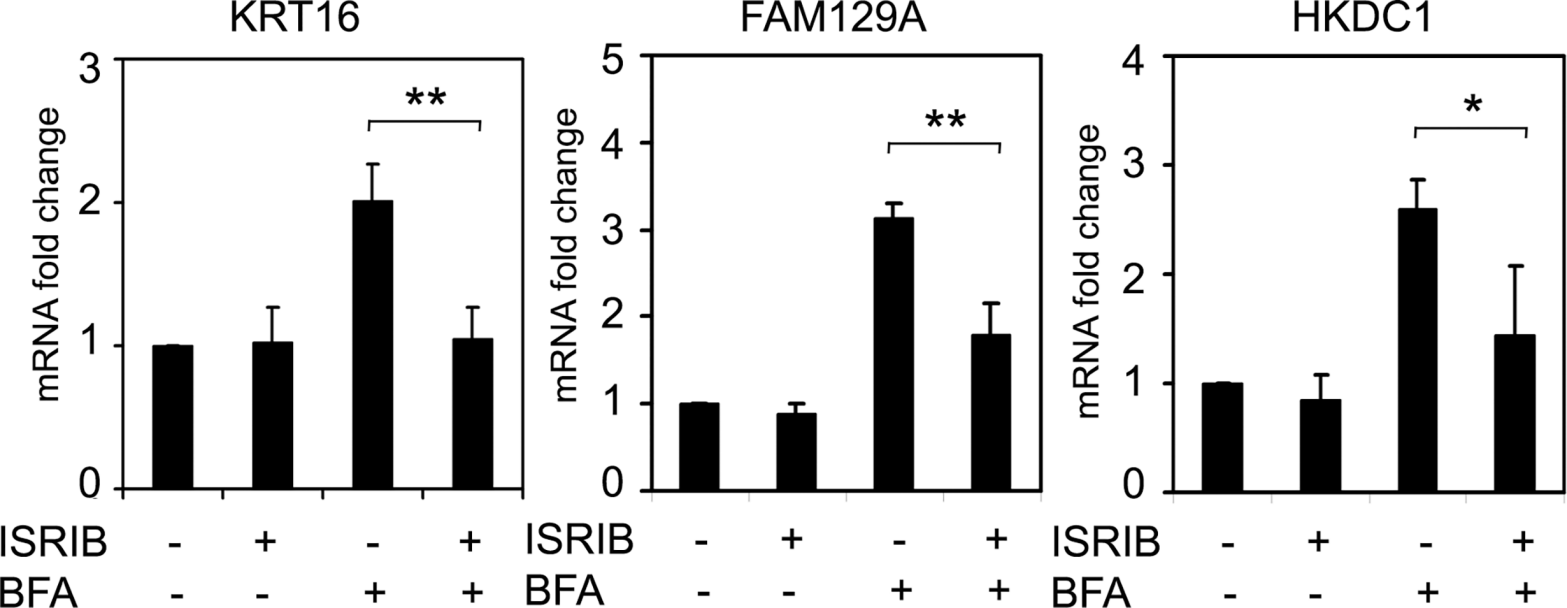
ISR is involved in the induction of KRT16, FAM129A and HKDC1 transcripts in response to ER stress. Fold changes of KRT16 (a), FAM129A (b) and HKDC1 (c) transcripts in HCT116 cells with or without ISRIB and treated with Brefeldin A (BFA) for 8 h as indicated. The data was obtained by RT-qPCR and processed as described in Materials and Methods.

We found that in HCT116 cells ISRIB can substantially inhibit the induction of KRT16, FAM129A and HKDC1 mRNAs by the ER stress inducer Brefeldin A (Fig 3). The results were reproduced in HeLa cells (S1 Fig). The data is in favor of a major role of the integrated stress response in the induction of *KRT16*, *FAM129A* and *HKDC1* genes during ER stress, presumably mediated by ATF4.

### Suppression of ATF4 mRNA by RNAi affects the induction of KRT16, FAM129A and HKDC1 transcripts

**Fig 4:**
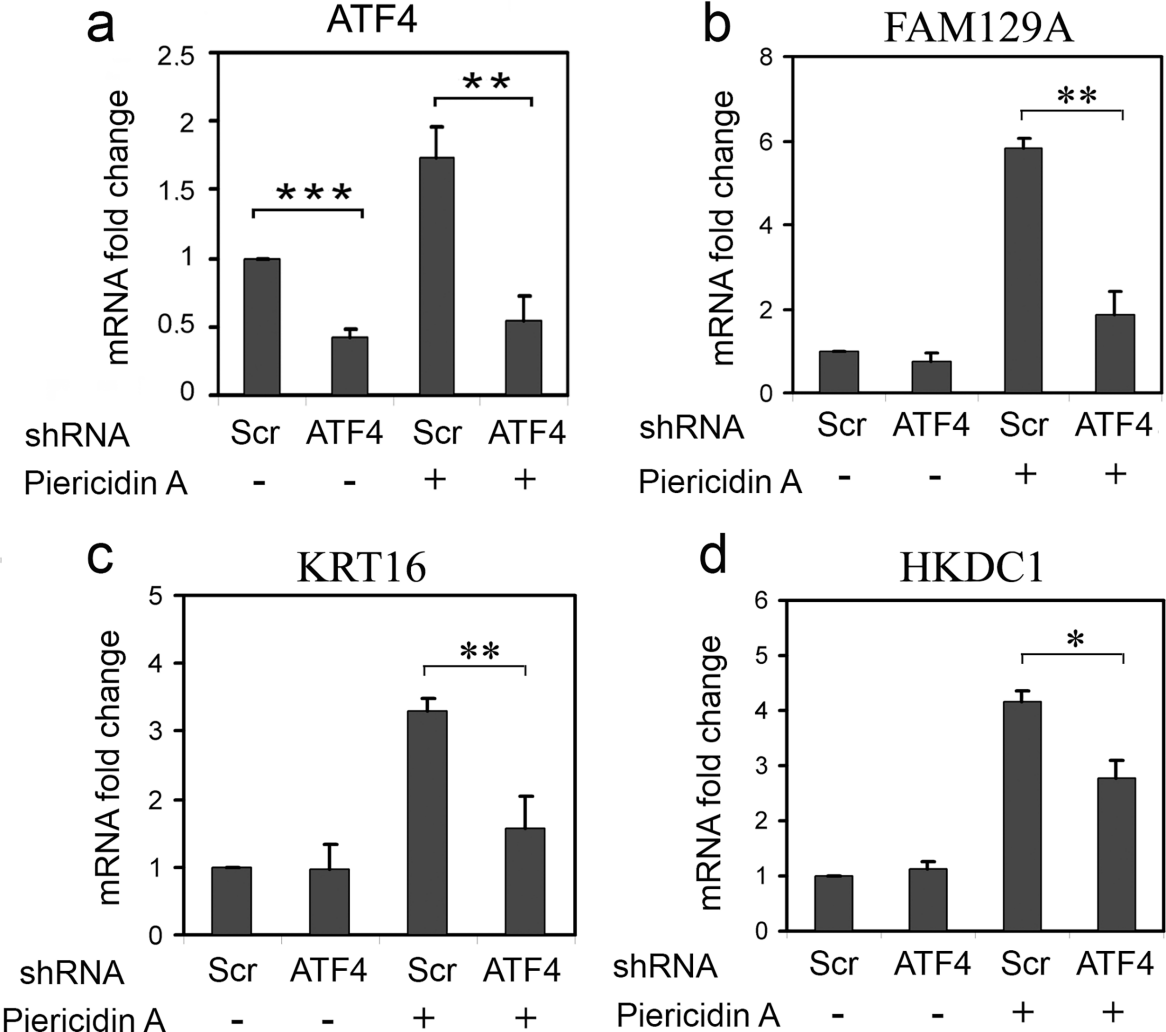
ATF4-specific RNAi leads to a suppression of *FAM129A*, *KRT16* and *HKDC1* induction in response to inhibition of the mitochondrial respiratory chain. Fold changes of ATF4 (a), FAM129A (b), KRT16 (c) and HKDC1 (d) transcripts in HCT116 cells expressing ATF4 shRNA (ATF4) or control scrambled shRNA (Scr) in response to treatment with Piericidin A for 8 h. The data was obtained by RT-qPCR and processed as described in Materials and Methods.

We inhibited transcripts from the ATF4 gene by stable transduction of a lentiviral construct expressing specific shRNA into HCT116 cells [18]. Levels of mRNAs from *ATF4*, *KRT16*, *FAM129A* and *HKDC1* genes were measured by real-time RT-PCR. There was a 2- fold suppression of the basal ATF4 mRNA level and a 3-fold suppression of the ATF4 mRNA induced by complex I inhibitor Piericidin A (Fig 4A). The suppression of ATF4 did not affect basal levels, but partially suppressed the Piericidin A induced activation of *FAM129A*, *KRT16* and *HKDC1* genes' transcription (Fig 4B–4D). This effect was more pronounced for *FAM129A*, but even though the reduction was more modest for *KRT16* and *HKDC1*, it was still statistically significant. The results indicate that ATF4 is involved in the upregulation of *FAM129A*, *KRT16* and *HKDC1* genes during the inhibition of the mitochondrial respiration chain.

**Fig 5:**
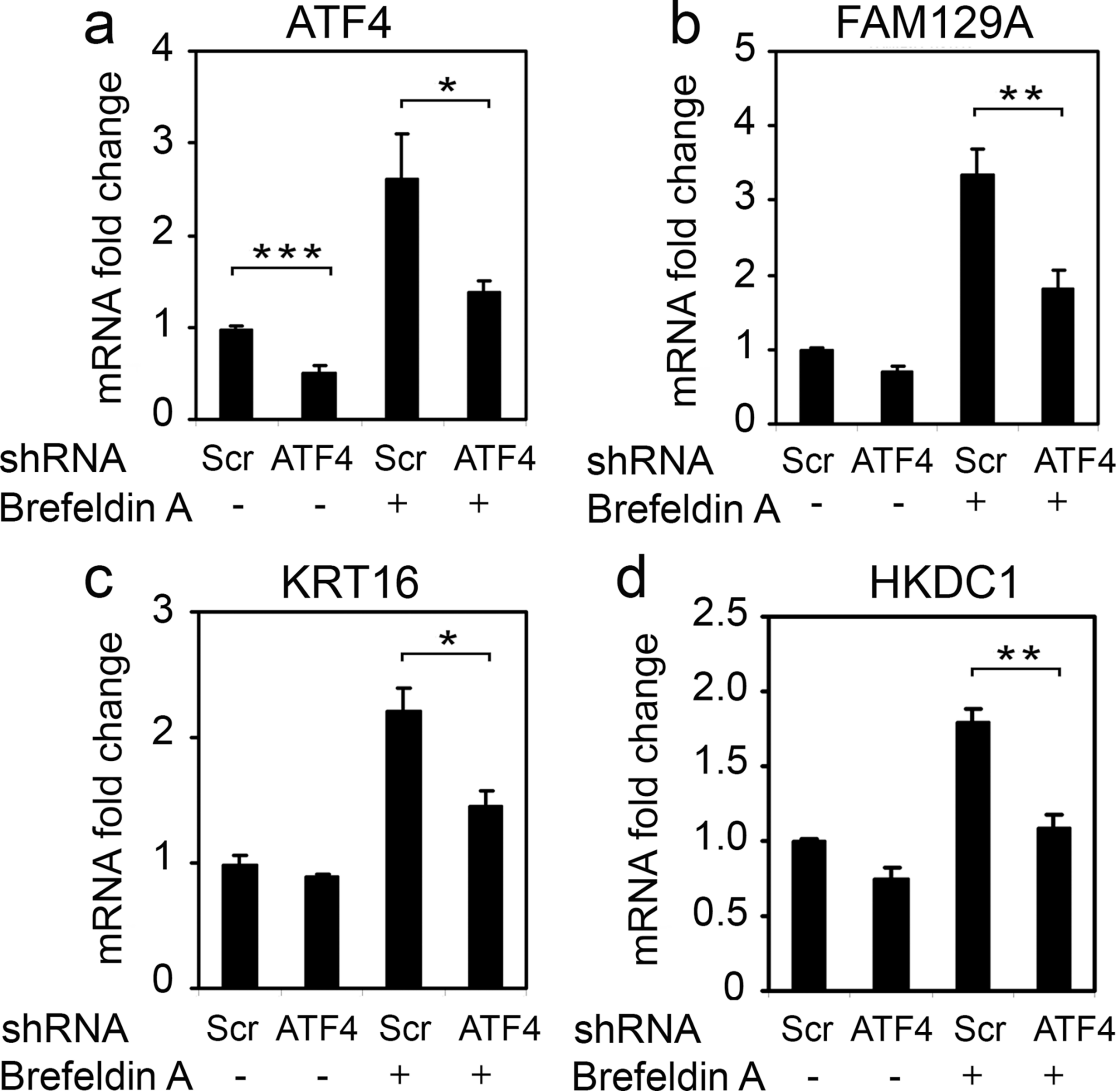
ATF4-specific RNAi leads to suppression of *FAM129A*, *KRT16* and *HKDC1* transcription activation in response to ER stress. Fold changes of ATF4 (a), FAM129A (b), KRT16 (c) and HKDC1 (d) transcripts in HCT116 cells expressing ATF4 shRNA (ATF4) or control scrambled shRNA (Scr) in response to treatment with Brefeldin A for 8 h. The data was obtained by RT-qPCR and processed as described in Materials and Methods.

There was also observed a partial suppression of FAM129A, KRT16 and HKDC1 mRNA induction in response to ER stress inducer Brefeldin A (Fig 5) further confirming the contribution of ATF4 in transcription regulation of the above genes during ER stress.

### An ectopic expression of ATF4 and ER stress affect protein levels of FAM129A/Niban protein

**Fig 6:**
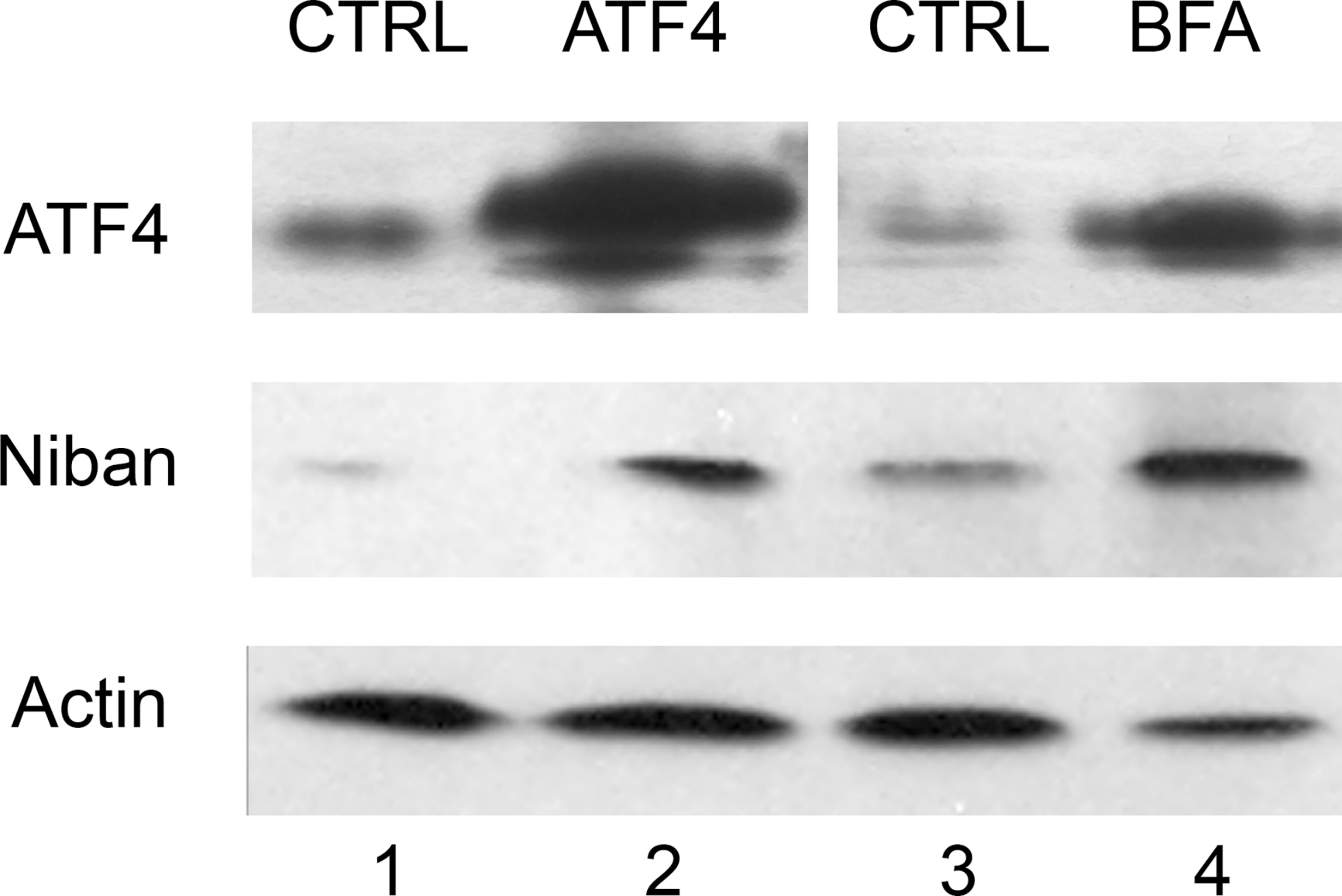
Ectopic overexpression of ATF4 and ER stress increase levels of Niban protein. Western analysis of ATF4 and Niban proteins in HCT116 cells overexpressing ATF4 from a plasmid construct (1, 2), or treated with Brifeldin A (BFA) for 16 hours (3, 4). Probing with actin antibodies was carried out as a loading control.

We overexpressed ATF4 protein in HCT116 cells from the introduced plasmid construct and measured protein levels by Western analysis. In parallel, we treated HCT116 cells with Brifeldin A for 16 hours and analyzed the protein extracts (Fig 6). There was a strong increase in ATF4 protein in the treated samples, as compared with the control. Antibodies to FAM129A/Niban protein also detected the increased expression of the protein in samples prepared from HCT116 cells overexpressing ATF4 or treated with Brifeldin A (Fig 6), as well as in HeLa cells treated with Brifeldin A and Tunicamycin (S2 Fig). The results indicate that the ATF4-medited upregulation of FAM129A/Niban also occurs at the protein level.

### ER stress induces the ATF4-mediated transcriptional upregulation of *FAM129A* and *HKDC1* genes but does not induce *KRT16* gene in transformed cell lines that were not derived from tumors

**Fig 7:**
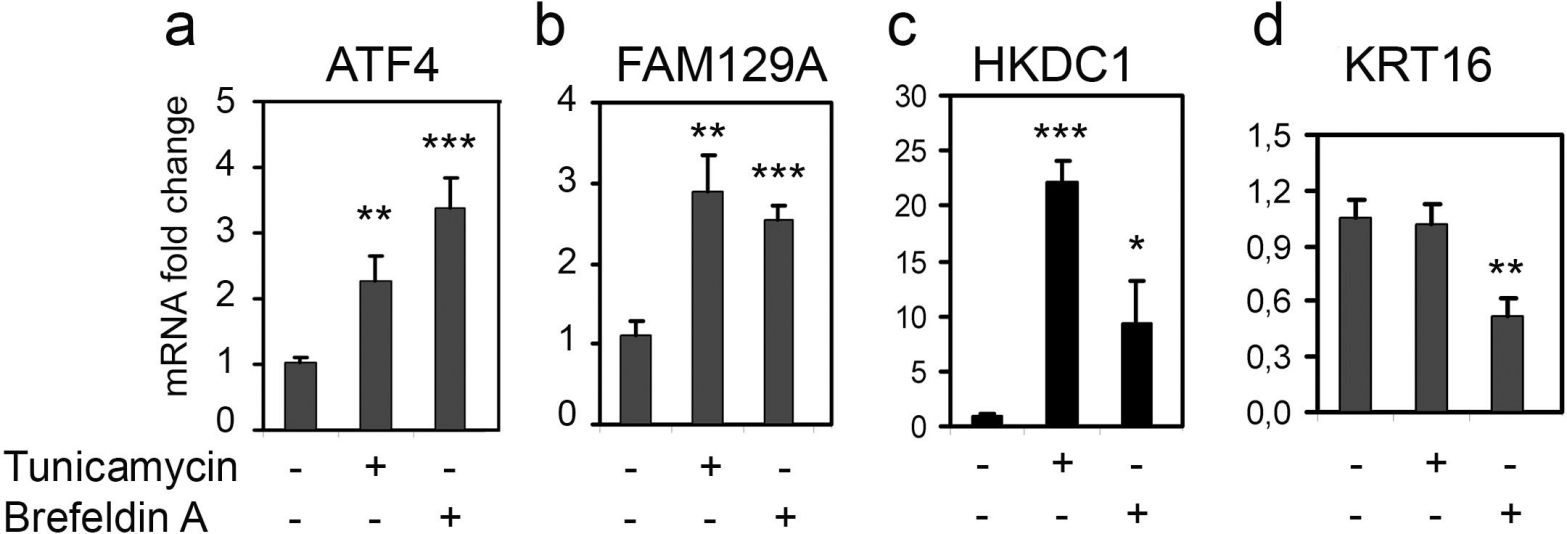
Impact of ER-stress on *ATF4*, *KRT16*, *FAM129A* and *HKDC1* genes expression in HaCaT cells. Fold changes of KRT16, FAM129A and HKDC1 transcripts in HaCaT cells treated with Brefeldin A or Tunicamycin for 15 h. The data was obtained by RT-qPCR and processed as described in Materials and Methods.

The experiments described above were performed in HCT116 colorectal carcinoma cells. In the cervical carcinoma HeLa cells *KRT16*, *FAM129A* and *HKDC1* transcripts were also induced in response to treatments associated with ATF4 upregulation (S3 Fig) and in response to ectopic overexpression of ATF4 [17]. To expand the observations we tested two additional cell lines. HaCaT cells, spontaneously transformed human skin keratinocyte cell line [26] was chosen as supposedly it represents a more suitable model for testing the regulation of cytokeratin-related KRT16 gene by ATF4. As expected, treatment of HaCaT cells with ER stress inducers Tunicamycin and Brefeldin A led to a statistically significant upregulation of ATF4 mRNA (Fig 7A) and a substantial induction of transcripts from *FAM129A* and *HKDC1* genes (Fig 7B and 7C). Surprisingly, there was no induction of transcripts from the *KRT16* gene, even a slight reduction in KRT16 mRNA level (Fig 7D). Similar results were obtained with the in vitro transformed human embryonic kidney cell line HEK293T (S4 Fig). The data obtained with these cell lines confirm the involvement of *FAM129A* and *HKDC1* genes in ISR-related responses mediated by ATF4 but raises questions about the controversial mode of regulation of *KRT16*.

### *KRT16* promoter is strongly induced by transcription factor ATF4

**Fig 8:**
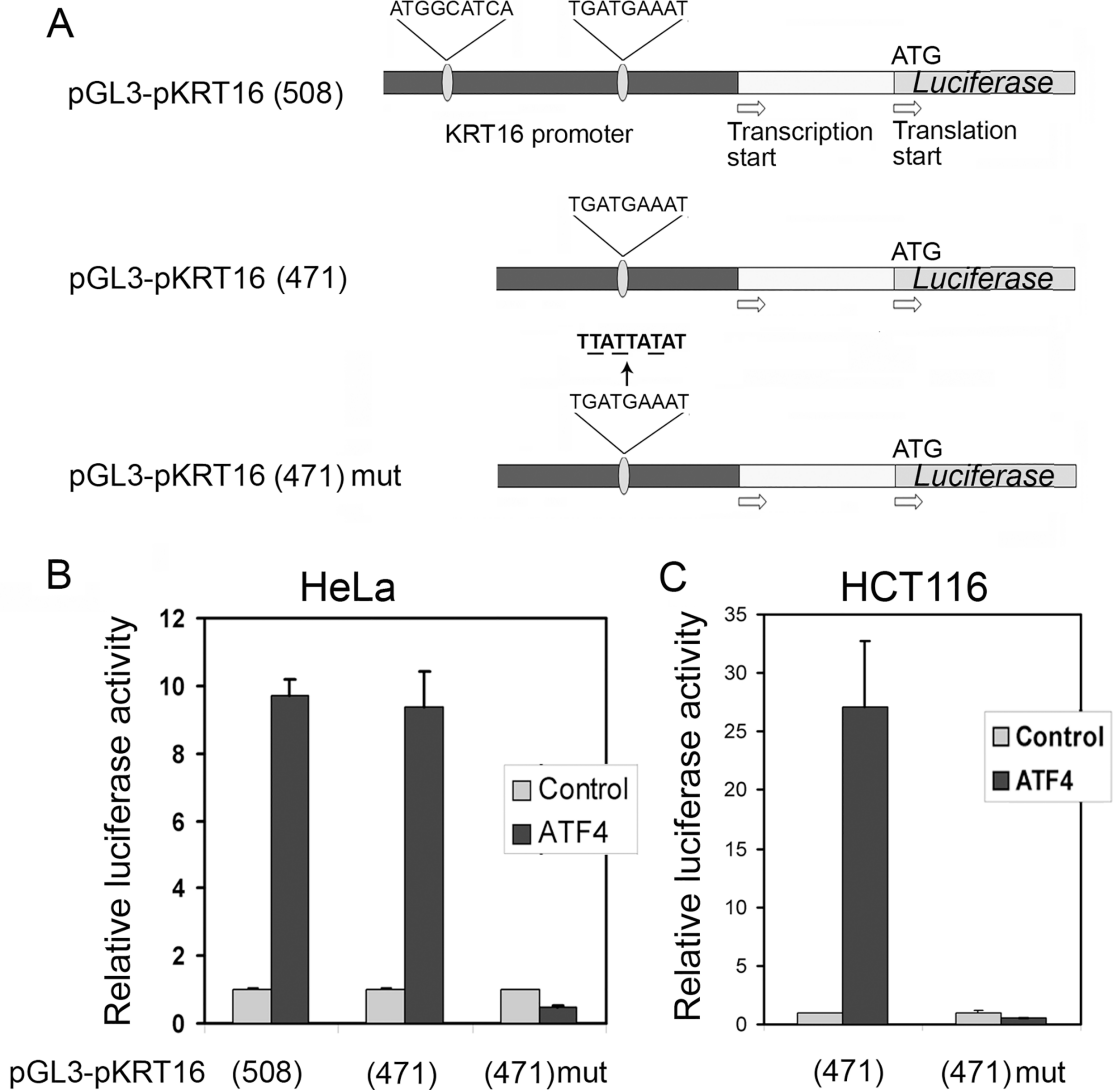
Ectopic expression of ATF4 induces transcription of KRT16 promoter-driven reporter gene due to the proximal putative ATF4 binding site. (A) The scheme of the reporter constructs engineered in this study. (B) Relative luciferase activity of reporters with both the distal and the proximal (pGL3-рKRT16(508)), only the proximal (pGL3-рKRT16(471)) or mutated proximal (pGL3-рKRT16(471)mut) putative ATF4-binding sites in transfected HeLa (B) or HCT116 (C) cells overexpressing ATF4 or the empty vector (Control). The reporter activities were normalized to those in the control cells.

To clarify a role of ATF4 in the regulation of *KRT16* promoter, we have constructed a reporter plasmid by placing firefly luciferase gene under control of the *KRT16* gene promoter. There are two potential ATF4 binding sites within the upstream region of *KRT16* gene, at position 171–163 and 482–474 upstream of the translation start site (Fig 8A), one (proximal) exactly matching the consensus sequence for CARE (C/EBP-ATF responsive element) and the other (distal) being different in only one nucleotide [4].

The 508 bp upstream region spanning from the translation start and composed of the promoter region comprising two putative ATF4-binding sites and the 5’-UTR, was amplified by PCR from total human DNA and inserted into pGL3-Basic vector upstream of firefly luciferase reporter gene. The obtained plasmid pGL3-рKRT16(508) was transfected into HeLa cells. A reporter plasmid expressing beta-galactosidase under control of the constitutive CMV promoter was used as normalization control. In response to overexpression of ATF4 we observed almost a 10-fold increase in activity of the reporter gene *luc* in the transfected cells (Fig 8B), indicating that ATF4 can activate KRT16 promoter. We also made a construct pGL3-рKRT16(471) containing only the proximal putative ATF4-responsive element by insertion of genomic fragment spanning from the upstream nucleotide 471 to translation start site (Fig 8A). These two constructs demonstrated similar induction of luciferase activity in response to the ectopic ATF4 expression (Fig 8B) suggesting that the distal site may not be involved into the ATF4-mediated regulation of KRT16 promoter. We then constructed and tested reporter plasmid pGL3-pKRT16 (471)-mut in which the proximal ATF4 binding site TGATGAAAT was mutated to inactive TTATTATAT sequence (Fig 8A). The mutations not only prevented the induction of a reporter gene in response to ectopic expression of ATF4, but also even led to a reliable 2-fold decrease in reporter activity below the basal levels (Fig 8B). The similar and even more pronounced effects were observed in transfected HCT116 cells (Fig 8C). We conclude that the promoter region of the *KRT16* gene does indeed contain the ATF4 responsive element that apparently is responsible for the upregulation of transcription as part of the ISR.

## Discussion

The results obtained in this study indicate that a dysfunction of mitochondrial respiration, as well as ER stress result in a partially ATF4-dependent stimulation of *KRT16*, *FAM129A* and *HKDC1* expression. The stimulation of the genes can be part of ISR, which is supported by the fact that a treatment of HCT116 cells with the specific inhibitor of the integrated stress response ISRIB resulted in a decrease of ER stress-induced levels of KRT16, FAM129A and HKDC1 transcripts. Similarly, the downregulation of ATF4 by RNAi in HCT116 cells has attenuated the induction of KRT16, FAM129A and HKDC1 mRNAs in response to ER stress or dysfunctional mitochondrial respiration. The same three genes demonstrate a transcriptional activation in response to ectopic expression of ATF4, and for FAM129A/Niban the upregulation was also observed at the protein level.

The similar mode of regulation of *KRT16*, *FAM129A* and *HKDC1* genes by ER stress and dysfunctional mitochondrial respiration was observed in cervical carcinoma HeLa cells. However, the regulation was different in two human transformed epithelial cell lines HaCaT and HEK293T that do not originate from patient-derived tumors. The HaCaT cells represent spontaneously transformed human keratinocytes [26], and HEK293T are human embryonic kidney cells transformed *in vitro* by oncogenes of adenovirus type 5 and SV40 [27]. In response to Tunicamycin and Brefeldin A in these cell lines we also observed the substantial upregulation of FAM129A and HKDC1 transcripts, but there was no effect or even a downregulation of KRT16 mRNA.

To confirm that *KRT16* gene is directly responding to ATF4 we tested luciferase reporter constructs containing upstream regions of the *KRT16* gene and identified a single active ATF4-specific DNA element TGATGAAAT located at a distance of 171 bp from the translation start. Identical ATF4 binding sites were previously identified in the gene for 4E-BP1 protein, a suppressor of the eukaryotic translation initiation factor eIF4E [4, 28] and the ATF4 mediated induction of 4E-BP1 was shown to protect insulin-producing pancreatic beta cells against ER stress [28].

Our results indicate that although *KRT16* gene contains a functional ATF4-responsive DNA element, its regulation by ATF4 is conditional, and in particular, the gene responds to ATF4 in the two patient-derived cancer cell lines, but not in the cell lines transformed *in vitro*. It is possible that the ATF4 regulation of *KRT16* is inhibited in normal cells, but is somehow activated during cancer progression processes. *KRT16* is not expressed in healthy epidermis, but the expression is associated with activation and hyperproliferation of keratinocytes [13]. The Keratin 16 protein participates in innate barrier function of the skin [29], and hereditary mutations in *KRT16* gene are associated with *pachyonychia congenita*, hyperkeratotic lesions of the skin, nails and oral epithelium [30]. KRT16 expression is upregulated in certain types of cancer [31–34]. Keratins form filamentous structures by a formation of heteropolymeric pair between type I (acidic) and type II (basic) molecules [35]. In stratified epithelia type-I KRT16 is co-expressed with the type-II KRT6 as the keratin pair, and both genes are coordinately induced by stresses, injuries and in pathologies [35]. However, we found that the upregulation of KRT16 mRNA by inducers of ER stress was not accompanied by the upregulation of KRT6A transcripts (S5 Fig). This result points to the potential non-canonical functions of KRT16 during stress responses.

Previous studies has identified a participation of several transcription factors that control KRT16 in response to different signals, such as stimulation with growth factors (EGF and TGFalpha) [36, 37], or oxidative stresses [38]. Here we show that *KRT16* gene is also linked to ISR through the action of ATF4. However, the participation of KRT16 in ISR seems to have a rather complex regulation. The failure to induce KRT16 mRNA in HaCaT and HEK293T cells, as opposed to the robust induction in HCT116 and HeLa cells may be explained by certain steps during cancer progression and selection of cells that are more competitive in tumors. The process may eliminate certain existing limitations that hinder the induction of *KRT16* transcription in response to ATF4. This hypothesis is substantiated by the reported upregulation of *KRT16* in tumors of the salivary glands [31], laryngeal [33], squamous skin [32] and breast cancer [34].

The two other genes identified here as ATF4-responsive members of ISR, *FAM129A* and *HKDC1*, were also implicated in adaptive processes and pathologies. FAM129A has previously been found to be inducible by ER stress [39], and here we show that levels of FAM129A transcripts and of Niban protein depend on ATF4. *FAM129A* encodes Niban protein that is overexpressed in many types of cancer. Niban can protect cells from apoptosis induced by genotoxic stress [15, 16, 40]. It positively regulates overall translation by affecting the phosphorylation of eIF2 alpha, S6 kinase and 4E-BP1 [39]. UV irradiation induces the phosphorylation of Niban by Akt protein kinase leading to an association of Niban with nucleophosmin NPM / B23 and dissociation of nucleophosmin from the complex with Mdm2 [41]. The released Mdm2 may stimulate the proteasomal degradation of p53 leading to an attenuated apoptotic response to stresses. In opposite, a suppression of Niban expression promotes the stabilization of p53 and increases apoptosis [41]. The two transcription factors ATF4 and p53 appear to act in an antagonistic manner. We found that p53 can suppress the induction of ATF4-dependent transcription during a sustained inhibition of mitochondrial Complex III [18]. In contrast, ATF4 can negatively regulate p53 by suppressing p14ARF, the inhibitor of the p53-Mdm2 interaction [42]. As mentioned above, Niban can also promote the Mdm2-assisted proteasome degradation of p53 [41]. Therefore, the ATF4 dependent upregulation of FAM129A can confer a pro-survival function during ISR, through the attenuation of p53-dependent apoptotic responses.

We identified *HKDC1* gene as a novel candidate component of ISR that responds to ER stress and dysfunctional respiration chain. The response only partially depends on ATF4 (Figs 3–5). Of note, in all cell lines studied the magnitude of HKDC1 mRNA upregulation by the inducers loosely correlated with the value of induction for transcripts from the ATF4 gene. So, unlike the ATF4 transcripts, the induction of HKDC1 mRNA by Tunicamycin was more prominent than by Brefeldin A in HaCaT (Fig 7A and 7C) and HeLa (S3 Fig) cells, suggesting that an additional signaling pathway may also be involved in regulation of HKDC1 transcription in response to ER stress. The possibility looks plausible as at least three known signaling pathways (PERK/eIF2α/ATF4, IRE1/XBP1 and ATF6) participate in the UPR/ER stress response [21, 22]. Details and specific role of IRE1/XBP1 and ATF6 pathways in the regulation needs to be further explored.

The human *HKDC1* gene encodes one of the five known hexokinases that phosphorylate hexose sugars [14, 43, 44]. Since phosphorylation is the first step of glucose catabolism, hexokinases may represent the critical step in regulation of energy metabolism that particularly limits the rate of cell growth and proliferation [45]. However, among the hexokinases only HKDC1 was shown to be dramatically overexpressed in the tumor tissues [14], which suggested a pivotal role of HKDC1 in cancer and particularly, a potentially promising therapeutic target for lung cancer [14]. Possibly, the upregulation of HKDC1 during ISR helps to overcome the deficiency of energy during the stress by stimulating the intracellular retention of glucose and driving it to glycolysis.

In conclusion, we obtained evidences for three novel components of ISR mediated by the ATF4 transcription factor and suggested their potential pro-survival roles during ER stress and dysfunctional mitochondrial respiration. However more functional data for each of the genes is required for the holistic understanding of their biological roles in homeostasis and response to stresses in normal and cancerous cells.

## Materials and methods

### Cell lines and treatments

HCT116, HeLa, HaCaT and HEK293T cell lines were cultured in DMEM, containing 10% fetal calf serum (HyClone) at 37^0^_C, 5% CO2 to 60–80% confluence.

The mitochondrial electron transfer chain inhibitors Myxothiazol or Piericidin A (Sigma-Aldrich, MO) were used at final concentrations of 1 and 2 μM, respectively; for induction of ER stress the cells were treated with 1 μg/ml Tunicamycin or 0.5 μg/ml Brefeldin A (Sigma-Aldrich, MO). ISRIB (Sigma-Aldrich, MO) was added to the final concentration 200 nM when indicated. The treatment time for each experiment is indicated in the legends to figures.

The lentiviral vectors expressing ATF4 shRNA or the control, scrambled shRNA, were prepared and introduced into HCT116 cell line as described [18].

The ectopic expression of ATF4 from transfected plasmid pHM-ATF4 was described in our recent report [17].

### Plasmid construction

For construction of the reporter plasmids containing two or one putative ATF4 binding sites, the DNA fragment (556 bp) comprising KRT16 promoter and 5’UTR was amplified by PCR on a total DNA from HeLa cell line with the primers KRT16_forward (5’-GTGGGACATGTGGG ATCCCCA-3’) and KRT16_reverse (5’-CATGACCACCTGCAGCCGCCAAGCTTAAT-3’). The PCR product was inserted into the plasmid pUC19 between BamHI and HindIII sites for sequencing, then recloned as BamHI-HindIII or SacI-HindIII fragments into the pGL3-Basic vector (Promega,WI) resulting in formation of pGL3-рKRT16(508) and pGL3-рKRT16(471) constructs, encoding 508 or 471 nucleotides upstream of KRT16 translation start codon, respectively.

The potential ATF4-binding site was inactivated by introducing three mutations by megaprimer PCR. The first PCR was performed with primers KRT16_forward and KRT16_mut_rev (5’- ACCGGGAGTTATTATATCCAGAGGGGAAC -3’), the plasmid pUC19 encoding KRT16 gene promoter and 5’-UTR was used as a template. The PCR product was utilized as a forward primer in the second PCR with the other primer KRT16_reverse. The PCR product was sequenced and recloned in pGL3-Basic vector as SacI-HindIII fragment forming the plasmid pGL3-рKRT16(471)mut.

### Reporter assays

HeLa and HCT116 cells were transfected with the reporter constructs as previously described [46]. The following plasmids were introduced into each well of the 12-well plate: one of the luciferase reporters, pGL3-pKRT16(508), pGL3- pKRT16 (471), or pGL3- pKRT16 (471)_mut (0.2 μg), the normalization plasmid pcDNA4/HisMax/lacZ (Invitrogen, CA) encoding β-galactosidase under control of the constitutive CMV promoter (0.5 μg) and the vector pHM-ATF4 (0.5 μg) for ATF4 ectopic expression [46]. The quantity of plasmid DNA was adjusted to 2 μg by the “empty” vector pcDNA4/HisMax/B used for pHM-ATF4 plasmid construction. The cells were lysed 44 h after transfection, the luciferase and β-galactosidase activities were measured as described previously [17]. Luciferase activity was normalized to β-galactosidase activity of the same lysate. For each measurement, at least three biological replicates were used.

### RT-qPCR

RNA isolation from the stressed and control cells, synthesis of cDNA, and real time PCR were performed as described [18]. Real-time PCR instrument CFX96 (Bio-Rad) and the following primers were used: KRT16_dir 5′-TGAGATGGAGCAGCAGAG-3’, KRT16_rev 5′- GACGA GGAGGAGGTGAAG-3’; FAM129A_dir 5′- AGGAGTCAGAGGAAGAGAAG-3’, FAM129A_rev 5′- GTTGCCACAGGATTCACC-3’; HKDC1_dir 5′- TTAAGGCACGAGGA GTTC-3’, HKDC1_rev 5′- TCATAGGCACAGGTCATC-3’; ATF4_dir 5′-CTTCACCTTCTTA CAACCTCTTC-3′, ATF4_rev 5′-GTAGTCTGGCTTCCTATCTCC-3′; 18S_dir 5′-CGGACAGGATTGACAGATTG-3′, 18S_rev 5′-CAGAGTCTCGTTCGTTATCG-3′.

The target ATF4, KRT16, FAM129A and HKDC1 transcripts were quantified using the reference 18 S rRNA for normalization.

### Western analysis

Western analysis of ATF4 and FAM129A/Niban proteins in HCT116 and HeLa cells was performed as described earlier [17, 19]. Antibodies to ATF4 (Abcam ab85049) and FAM129A/Niban (LifeSpan BioSciences Inc., LS-C80523) and actin (Santa-Cruz Biotech, Inc. C-2, sc8432) were diluted at a ratio of 1:500.

### Statistical analysis

Statistical analysis was performed using online t-test calculator of GraphPad Software (https://www.graphpad.com/quickcalcs/ttest1.cfm). Data are expressed as mean ± SEM from at least three independent experiments. Mean values were compared statistically using unpaired Student's *t*-test. The following marks were used for P-values <0.05 (*), <0.01 (**), <0.001 (***).

## Supporting information

S1 Fig ISR is involved in the induction of FAM129A, KRT16 and HKDC1 transcripts in HeLa cells in response to ER stress. Fold changes of FAM129A, KRT16 and HKDC1 transcripts in HeLa cells with or without ISRIB and treated with Brefeldin A (BFA) for 8 h (for FAM129A, KRT16) or 16 h (for HKDC1) as indicated. The data were obtained by RT-qPCR and processed as described in Materials and Methods.
(DOCX)

S2 Fig Upregulation of Niban protein by ER stress in HeLa cells. HeLa cells were treated for 16 hours with Brifeldin A (BFA) or Tunicamycin (Tm) and subjected to Western analysis with antibodies to ATF4 (a) or Niban (a,b) proteins. Probing with actin antibodies was carried out as a loading control.
(DOCX)

S3 Fig Induction of ATF4, KRT16, FAM129A and HKDC1 transcripts by ER stress or inhibition of mitochondrial respiratory chain in HeLa cells. Fold changes of ATF4, KRT16, FAM129A and HKDC1 transcripts in HeLa cells treated with Tunicamycin (Tm) for 14h, Brefeldin A (BFA) or Piericidin A (Pier) for 8h. The data was obtained by RT-qPCR and processed as described in Materials and Methods.
(DOCX)

S4 Fig Induction of ATF4, KRT16, FAM129A and HKDC1 transcripts by ER stress or inhibition of mitochondrial respiratory chain in HEK293T cells. Fold changes of ATF4, KRT16, FAM129A and HKDC1 transcripts in HEK293T cells treated with Tunicamycin (Tm), Brefeldin A (BFA) or Piericidin A (Pier) for 14 h. The data was obtained by RT-qPCR and processed as described in Materials and Methods.
(DOCX)

S5 Fig Levels of KRT6A mRNA in HCT116 cells treated with ER stress inducers. Fold changes of KRT6A transcripts in HCT116 cells treated with Tunicamycin (Tm) or Brefeldin A (BFA) for 14 h. The data was obtained by RT-qPCR and processed as described in Materials and Methods.
(DOCX)

## References

[1] Young SK, Wek RC. Upstream Open Reading Frames Differentially Regulate Gene-specific Translation in the Integrated Stress Response. J Biol Chem. 2016;291(33):16927–35. Epub 2016/07/01. doi: 10.1074/jbc.R116.733899 ; PubMed Central PMCID: PMCPMC5016099. 27358398 10.1074/jbc.R116.733899 PMC5016099

[2] Baird TD, Wek RC. Eukaryotic initiation factor 2 phosphorylation and translational control in metabolism. Adv Nutr. 2012;3(3):307–21. Epub 2012/05/16. doi: 10.3945/an.112.002113 ; PubMed Central PMCID: PMCPmc3649462. 22585904 10.3945/an.112.002113 PMC3649462

[3] Harding HP, Zhang Y, Zeng H, Novoa I, Lu PD, Calfon M, et al An integrated stress response regulates amino acid metabolism and resistance to oxidative stress. Mol Cell. 2003;11(3):619–33. Epub 2003/04/02. . 12667446 10.1016/s1097-2765(03)00105-9

[4] Kilberg MS, Shan J, Su N. ATF4-dependent transcription mediates signaling of amino acid limitation. Trends Endocrinol Metab. 2009;20(9):436–43. Epub 2009/10/06. doi: 10.1016/j.tem.2009.05.008 ; PubMed Central PMCID: PMCPMC3587693. 19800252 10.1016/j.tem.2009.05.008 PMC3587693

[5] Han J, Back SH, Hur J, Lin YH, Gildersleeve R, Shan J, et al ER-stress-induced transcriptional regulation increases protein synthesis leading to cell death. Nat Cell Biol. 2013;15(5):481–90. Epub 2013/04/30. doi: 10.1038/ncb2738 ; PubMed Central PMCID: PMCPMC3692270. 23624402 10.1038/ncb2738 PMC3692270

[6] Huang ML-H, Sivagurunathan S, Ting S, Jansson PJ, Austin CJD, Kelly MA, et al Molecular and functional alterations in a mouse cardiac model of Friedreich ataxia: activation of the integrated stress response, eIF2α phosphorylation, and the induction of downstream targets. The American journal of pathology. 2013;183(3):745–57. doi: 10.1016/j.ajpath.2013.05.032 . 23886890 10.1016/j.ajpath.2013.05.032

[7] Ohno M. Roles of eIF2alpha kinases in the pathogenesis of Alzheimer's disease. Frontiers in molecular neuroscience. 2014;7:22 Epub 2014/05/06. doi: 10.3389/fnmol.2014.00022 ; PubMed Central PMCID: PMCPMC3997008. 24795560 10.3389/fnmol.2014.00022 PMC3997008

[8] Sun X, Liu J, Crary JF, Malagelada C, Sulzer D, Greene LA, et al ATF4 protects against neuronal death in cellular Parkinson's disease models by maintaining levels of parkin. J Neurosci. 2013;33(6):2398–407. Epub 2013/02/09. doi: 10.1523/JNEUROSCI.2292-12.2013 ; PubMed Central PMCID: PMCPMC3711618. 23392669 10.1523/JNEUROSCI.2292-12.2013 PMC3711618

[9] van 't Wout EF, Hiemstra PS, Marciniak SJ. The integrated stress response in lung disease. American journal of respiratory cell and molecular biology. 2014;50(6):1005–9. Epub 2014/03/13. doi: 10.1165/rcmb.2014-0019TR . 24605820 10.1165/rcmb.2014-0019TR

[10] Chiang CK, Hsu SP, Wu CT, Huang JW, Cheng HT, Chang YW, et al Endoplasmic reticulum stress implicated in the development of renal fibrosis. Mol Med. 2011;17(11–12):1295–305. Epub 2011/08/25. doi: 10.2119/molmed.2011.00131 ; PubMed Central PMCID: PMCPMC3324175. 21863214 10.2119/molmed.2011.00131 PMC3324175

[11] Singleton DC, Harris AL. Targeting the ATF4 pathway in cancer therapy. Expert Opin Ther Targets. 2012;16(12):1189–202. Epub 2012/09/27. doi: 10.1517/14728222.2012.728207 . 23009153 10.1517/14728222.2012.728207

[12] Dey S, Baird TD, Zhou D, Palam LR, Spandau DF, Wek RC. Both transcriptional regulation and translational control of ATF4 are central to the integrated stress response. J Biol Chem. 2010;285(43):33165–74. Epub 2010/08/25. doi: 10.1074/jbc.M110.167213 ; PubMed Central PMCID: PMCPMC2963398. 20732869 10.1074/jbc.M110.167213 PMC2963398

[13] Machesney M, Tidman N, Waseem A, Kirby L, Leigh I. Activated keratinocytes in the epidermis of hypertrophic scars. Am J Pathol. 1998;152(5):1133–41. Epub 1998/05/20. ; PubMed Central PMCID: PMCPMC1858601. 9588880 PMC1858601

[14] Li GH, Huang JF. Inferring therapeutic targets from heterogeneous data: HKDC1 is a novel potential therapeutic target for cancer. Bioinformatics. 2014;30(6):748–52. Epub 2013/10/29. doi: 10.1093/bioinformatics/btt606 . 24162464 10.1093/bioinformatics/btt606

[15] Matsumoto F, Fujii H, Abe M, Kajino K, Kobayashi T, Matsumoto T, et al A novel tumor marker, Niban, is expressed in subsets of thyroid tumors and Hashimoto's thyroiditis. Hum Pathol. 2006;37(12):1592–600. Epub 2006/09/05. doi: 10.1016/j.humpath.2006.06.022 . 16949643 10.1016/j.humpath.2006.06.022

[16] Carvalheira G, Nozima BH, Cerutti JM. microRNA-106b-mediated down-regulation of C1orf24 expression induces apoptosis and suppresses invasion of thyroid cancer. Oncotarget. 2015;6(29):28357–70. Epub 2015/09/01. doi: 10.18632/oncotarget.4947 ; PubMed Central PMCID: PMCPMC4695065. 26317551 10.18632/oncotarget.4947 PMC4695065

[17] Kovaleva IE, Garaeva AA, Chumakov PM, Evstafieva AG. Intermedin/Adrenomedullin 2 is a stress-inducible gene controlled by Activating Transcription Factor 4. Gene. 2016;590:177–85. doi: 10.1016/j.gene.2016.06.037 27328454 10.1016/j.gene.2016.06.037

[18] Evstafieva AG, Garaeva AA, Khutornenko AA, Klepikova AV, Logacheva MD, Penin AA, et al A sustained deficiency of mitochondrial respiratory complex III induces an apoptotic cell death through the p53-mediated inhibition of pro-survival activities of the activating transcription factor 4. Cell death & disease. 2014;5:e1511 Epub 2014/11/07. doi: 10.1038/cddis.2014.469 . 25375376 10.1038/cddis.2014.469 PMC4260727

[19] Khutornenko AA, Dalina AA, Chernyak BV, Chumakov PM, Evstafieva AG. The Role of Dihydroorotate Dehydrogenase in Apoptosis Induction in Response to Inhibition of the Mitochondrial Respiratory Chain Complex III. Acta naturae. 2014;6(1):69–75. Epub 2014/04/29. ; PubMed Central PMCID: PMCPMC3999468. 24772329 PMC3999468

[20] Khutornenko AA, Roudko VV, Chernyak BV, Vartapetian AB, Chumakov PM, Evstafieva AG. Pyrimidine biosynthesis links mitochondrial respiration to the p53 pathway. Proc Natl Acad Sci U S A. 2010;107(29):12828–33. Epub 2010/06/23. doi: 10.1073/pnas.0910885107 ; PubMed Central PMCID: PMC2919937. 20566882 10.1073/pnas.0910885107 PMC2919937

[21] Wek RC, Cavener DR. Translational control and the unfolded protein response. Antioxid Redox Signal. 2007;9(12):2357–71. Epub 2007/09/01. doi: 10.1089/ars.2007.1764 . 17760508 10.1089/ars.2007.1764

[22] Sykes EK, Mactier S, Christopherson RI. Melanoma and the Unfolded Protein Response. Cancers. 2016;8(3). Epub 2016/03/02. doi: 10.3390/cancers8030030 ; PubMed Central PMCID: PMCPMC4810114. 26927180 10.3390/cancers8030030 PMC4810114

[23] Sidrauski C, Acosta-Alvear D, Khoutorsky A, Vedantham P, Hearn BR, Li H, et al Pharmacological brake-release of mRNA translation enhances cognitive memory. Elife. 2013;2:e00498 Epub 2013/06/07. doi: 10.7554/eLife.00498 ; PubMed Central PMCID: PMCPMC3667625. 23741617 10.7554/eLife.00498 PMC3667625

[24] Sekine Y, Zyryanova A, Crespillo-Casado A, Fischer PM, Harding HP, Ron D. Stress responses. Mutations in a translation initiation factor identify the target of a memory-enhancing compound. Science. 2015;348(6238):1027–30. Epub 2015/04/11. doi: 10.1126/science.aaa6986 ; PubMed Central PMCID: PMCPmc4538794. 25858979 10.1126/science.aaa6986 PMC4538794

[25] Sidrauski C, McGeachy AM, Ingolia NT, Walter P. The small molecule ISRIB reverses the effects of eIF2alpha phosphorylation on translation and stress granule assembly. Elife. 2015;4:e05033 Epub 2015/02/27. doi: 10.7554/eLife.05033 ; PubMed Central PMCID: PMCPmc4341466. 25719440 10.7554/eLife.05033 PMC4341466

[26] Boukamp P, Petrussevska RT, Breitkreutz D, Hornung J, Markham A, Fusenig NE. Normal keratinization in a spontaneously immortalized aneuploid human keratinocyte cell line. J Cell Biol. 1988;106(3):761–71. Epub 1988/03/01. ; PubMed Central PMCID: PMCPMC2115116. 2450098 10.1083/jcb.106.3.761 PMC2115116

[27] DuBridge RB, Tang P, Hsia HC, Leong PM, Miller JH, Calos MP. Analysis of mutation in human cells by using an Epstein-Barr virus shuttle system. Mol Cell Biol. 1987;7(1):379–87. Epub 1987/01/01. ; PubMed Central PMCID: PMCPMC365079. 3031469 10.1128/mcb.7.1.379-387.1987 PMC365079

[28] Yamaguchi S, Ishihara H, Yamada T, Tamura A, Usui M, Tominaga R, et al ATF4-mediated induction of 4E-BP1 contributes to pancreatic beta cell survival under endoplasmic reticulum stress. Cell Metab. 2008;7(3):269–76. Epub 2008/03/05. doi: 10.1016/j.cmet.2008.01.008 . 18316032 10.1016/j.cmet.2008.01.008

[29] Lessard JC, Pina-Paz S, Rotty JD, Hickerson RP, Kaspar RL, Balmain A, et al Keratin 16 regulates innate immunity in response to epidermal barrier breach. Proc Natl Acad Sci U S A. 2013;110(48):19537–42. Epub 2013/11/13. doi: 10.1073/pnas.1309576110 ; PubMed Central PMCID: PMCPMC3845144. 24218583 10.1073/pnas.1309576110 PMC3845144

[30] Eliason MJ, Leachman SA, Feng BJ, Schwartz ME, Hansen CD. A review of the clinical phenotype of 254 patients with genetically confirmed pachyonychia congenita. Journal of the American Academy of Dermatology. 2012;67(4):680–6. Epub 2012/01/24. doi: 10.1016/j.jaad.2011.12.009 . 22264670 10.1016/j.jaad.2011.12.009

[31] Bell D, Bell AH, Bondaruk J, Hanna EY, Weber RS. In-depth characterization of the salivary adenoid cystic carcinoma transcriptome with emphasis on dominant cell type. Cancer. 2016;122(10):1513–22. Epub 2016/03/10. doi: 10.1002/cncr.29959 . 26953815 10.1002/cncr.29959

[32] Hameetman L, Commandeur S, Bavinck JN, Wisgerhof HC, de Gruijl FR, Willemze R, et al Molecular profiling of cutaneous squamous cell carcinomas and actinic keratoses from organ transplant recipients. BMC cancer. 2013;13:58 Epub 2013/02/06. doi: 10.1186/1471-2407-13-58 ; PubMed Central PMCID: PMCPMC3570297. 23379751 10.1186/1471-2407-13-58 PMC3570297

[33] Zha C, Jiang XH, Peng SF. iTRAQ-based quantitative proteomic analysis on S100 calcium binding protein A2 in metastasis of laryngeal cancer. PLoS One. 2015;10(4):e0122322 Epub 2015/04/16. doi: 10.1371/journal.pone.0122322 ; PubMed Central PMCID: PMCPMC4395276. 25874882 10.1371/journal.pone.0122322 PMC4395276

[34] Yu KD, Zhu R, Zhan M, Rodriguez AA, Yang W, Wong S, et al Identification of prognosis-relevant subgroups in patients with chemoresistant triple-negative breast cancer. Clin Cancer Res. 2013;19(10):2723–33. Epub 2013/04/04. doi: 10.1158/1078-0432.CCR-12-2986 ; PubMed Central PMCID: PMCPMC3655097. 23549873 10.1158/1078-0432.CCR-12-2986 PMC3655097

[35] Moll R, Divo M, Langbein L. The human keratins: biology and pathology. Histochemistry and cell biology. 2008;129(6):705–33. Epub 2008/05/08. doi: 10.1007/s00418-008-0435-6 ; PubMed Central PMCID: PMCPMC2386534. 18461349 10.1007/s00418-008-0435-6 PMC2386534

[36] Wang YN, Chang WC. Induction of disease-associated keratin 16 gene expression by epidermal growth factor is regulated through cooperation of transcription factors Sp1 and c-Jun. J Biol Chem. 2003;278(46):45848–57. Epub 2003/09/05. doi: 10.1074/jbc.M302630200 . 12954631 10.1074/jbc.M302630200

[37] Wang YN, Chen YJ, Chang WC. Activation of extracellular signal-regulated kinase signaling by epidermal growth factor mediates c-Jun activation and p300 recruitment in keratin 16 gene expression. Molecular pharmacology. 2006;69(1):85–98. Epub 2005/10/11. doi: 10.1124/mol.105.016220 . 16214953 10.1124/mol.105.016220

[38] Endo H, Sugioka Y, Nakagi Y, Saijo Y, Yoshida T. A novel role of the NRF2 transcription factor in the regulation of arsenite-mediated keratin 16 gene expression in human keratinocytes. Environmental health perspectives. 2008;116(7):873–9. Epub 2008/07/17. doi: 10.1289/ehp.10696 ; PubMed Central PMCID: PMCPMC2453154. 18629308 10.1289/ehp.10696 PMC2453154

[39] Sun GD, Kobayashi T, Abe M, Tada N, Adachi H, Shiota A, et al The endoplasmic reticulum stress-inducible protein Niban regulates eIF2alpha and S6K1/4E-BP1 phosphorylation. Biochem Biophys Res Commun. 2007;360(1):181–7. Epub 2007/06/26. doi: 10.1016/j.bbrc.2007.06.021 . 17588536 10.1016/j.bbrc.2007.06.021

[40] Ito S, Fujii H, Matsumoto T, Abe M, Ikeda K, Hino O. Frequent expression of Niban in head and neck squamous cell carcinoma and squamous dysplasia. Head Neck. 2010;32(1):96–103. Epub 2009/06/19. doi: 10.1002/hed.21153 . 19536772 10.1002/hed.21153

[41] Ji H, Ding Z, Hawke D, Xing D, Jiang BH, Mills GB, et al AKT-dependent phosphorylation of Niban regulates nucleophosmin- and MDM2-mediated p53 stability and cell apoptosis. EMBO Rep. 2012;13(6):554–60. Epub 2012/04/19. doi: 10.1038/embor.2012.53 ; PubMed Central PMCID: PMCPMC3367238. 22510990 10.1038/embor.2012.53 PMC3367238

[42] Horiguchi M, Koyanagi S, Hamdan AM, Kakimoto K, Matsunaga N, Yamashita C, et al Rhythmic control of the ARF-MDM2 pathway by ATF4 underlies circadian accumulation of p53 in malignant cells. Cancer Res. 2013;73(8):2639–49. Epub 2013/04/13. doi: 10.1158/0008-5472.CAN-12-2492 . 23580573 10.1158/0008-5472.CAN-12-2492

[43] Irwin DM, Tan H. Molecular evolution of the vertebrate hexokinase gene family: Identification of a conserved fifth vertebrate hexokinase gene. Comparative biochemistry and physiology Part D, Genomics & proteomics. 2008;3(1):96–107. Epub 2008/03/01. doi: 10.1016/j.cbd.2007.11.002 . 20483211 10.1016/j.cbd.2007.11.002

[44] Guo C, Ludvik AE, Arlotto ME, Hayes MG, Armstrong LL, Scholtens DM, et al Coordinated regulatory variation associated with gestational hyperglycaemia regulates expression of the novel hexokinase HKDC1. Nature communications. 2015;6:6069 Epub 2015/02/05. doi: 10.1038/ncomms7069 ; PubMed Central PMCID: PMCPMC4318120. 25648650 10.1038/ncomms7069 PMC4318120

[45] Olovnikov IA, Kravchenko JE, Chumakov PM. Homeostatic functions of the p53 tumor suppressor: regulation of energy metabolism and antioxidant defense. Seminars in cancer biology. 2009;19(1):32–41. Epub 2008/12/23. S1044-579X(08)00109-0 [pii] doi: 10.1016/j.semcancer.2008.11.005 ; PubMed Central PMCID: PMC2646792. 19101635 10.1016/j.semcancer.2008.11.005 PMC2646792

[46] Garaeva AA, Kovaleva IE, Chumakov PM, Evstaf'eva AG. Mitochondrial dysfunction induces SESN2 gene expression through the Activating Transcription Factor 4. Cell Cycle. 2016;15(1):64–71. doi: 10.1080/15384101.2015.1120929 26771712 10.1080/15384101.2015.1120929 PMC4825760

