# Supplementary material for: Implication of KRT16, FAM129A and HKDC1 genes as ATF4 regulated components of the integrated stress response
Source: PLoS One. 2018 Feb 8;13(2):e0191107. doi: 10.1371/journal.pone.0191107 (PMC5805170; doi:10.1371/journal.pone.0191107)
Supplement: S1 Fig — Fold changes of FAM129A, KRT16 and HKDC1 transcripts in HeLa cells with or without ISRIB and treated with Brefeldin A (BFA) for 8 h (for FAM129A, KRT16) or 16 h (for HKDC1) as indicated. The data were obtained by RT-qPCR and processed as described in Materials and Methods. (DOCX) [file pone.0191107.s001.docx]

Supporting information Fig S1


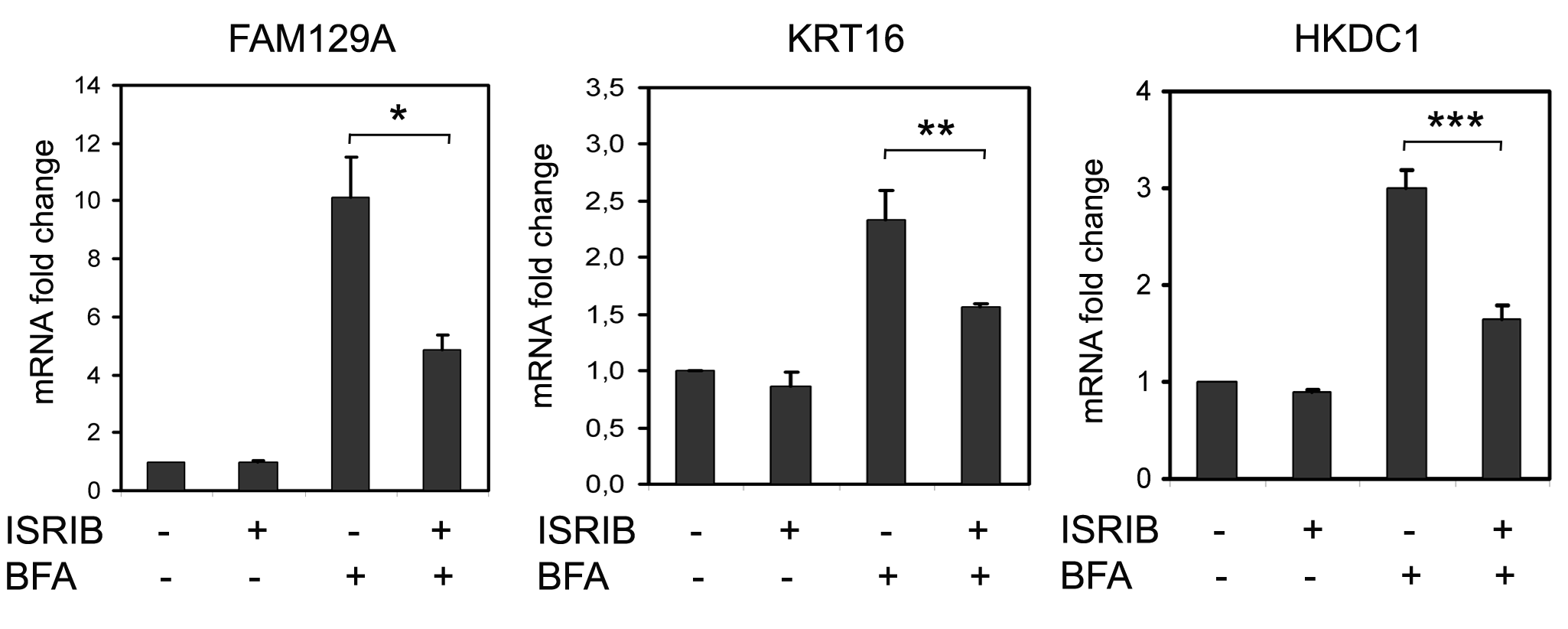


**Fig S1. ISR is involved in the induction of FAM129A, KRT16 and HKDC1 transcripts in HeLa cells in response to ER stress.** Fold changes of FAM129A, KRT16 and HKDC1 transcripts in HeLa cells with or without ISRIB and treated with Brefeldin A (BFA) for 8 h (for FAM129A, KRT16) or 16 h (for HKDC1) as indicated. The data were obtained by RT-qPCR and processed as described in Materials and Methods.
