# Supplementary material for: Implication of KRT16, FAM129A and HKDC1 genes as ATF4 regulated components of the integrated stress response
Source: PLoS One. 2018 Feb 8;13(2):e0191107. doi: 10.1371/journal.pone.0191107 (PMC5805170; doi:10.1371/journal.pone.0191107)
Supplement: S2 Fig — HeLa cells were treated for 16 hours with Brifeldin A (BFA) or Tunicamycin (Tm) and subjected to Western analysis with antibodies to ATF4 (a) or Niban (a,b) proteins. Probing with actin antibodies was carried out as a loading control. (DOCX) [file pone.0191107.s002.docx]

Supporting information Fig S2


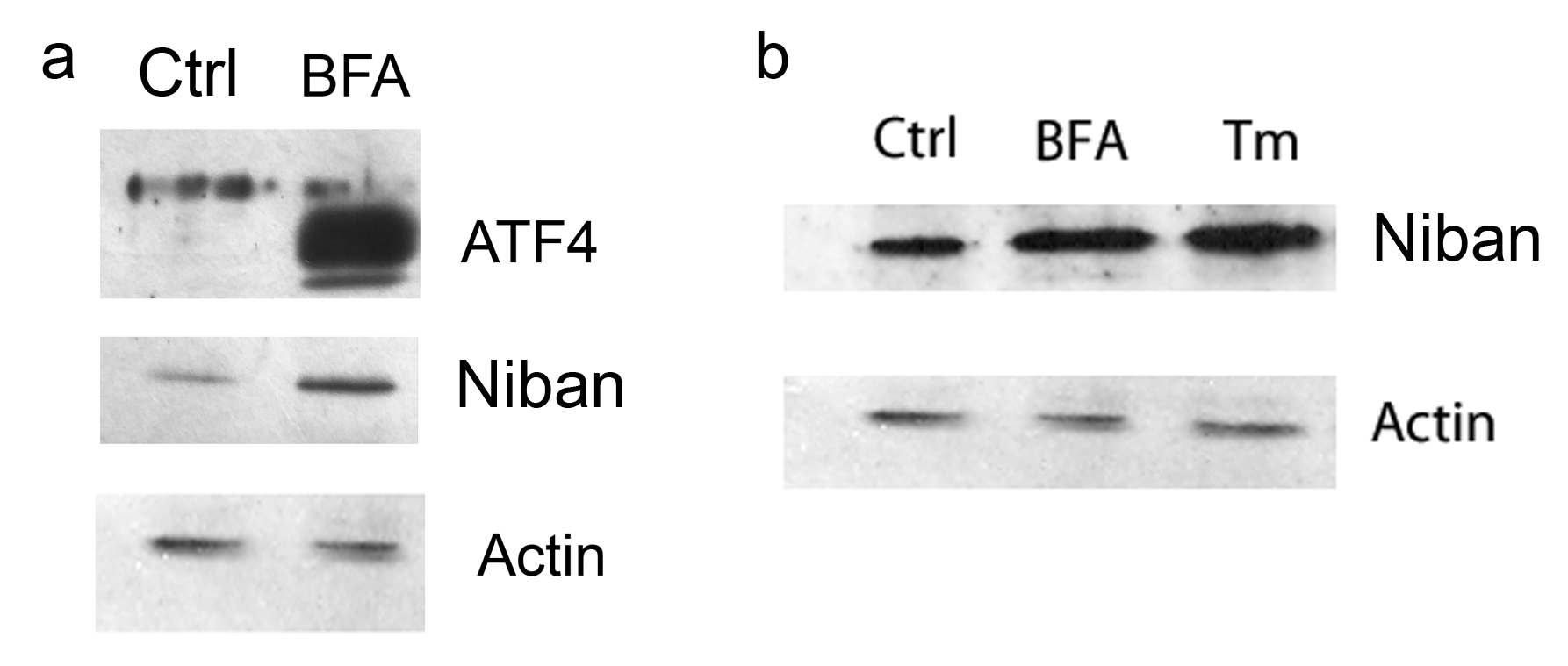


**Fig S2. Upregulation of Niban protein by ER stress in HeLa cells.**HeLa cells were treated for 16 hours with Brifeldin A (BFA) or Tunicamycin (Tm) and subjected to Western analysis with antibodies to ATF4 (a) or Niban (a,b) proteins. Probing with actin antibodies was carried out as a loading control.
