# Supplementary material for: Implication of KRT16, FAM129A and HKDC1 genes as ATF4 regulated components of the integrated stress response
Source: PLoS One. 2018 Feb 8;13(2):e0191107. doi: 10.1371/journal.pone.0191107 (PMC5805170; doi:10.1371/journal.pone.0191107)
Supplement: S3 Fig — Fold changes of ATF4, KRT16, FAM129A and HKDC1 transcripts in HeLa cells treated with Tunicamycin (Tm) for 14h, Brefeldin A (BFA) or Piericidin A (Pier) for 8h. The data was obtained by RT-qPCR and processed as described in Materials and Methods. (DOCX) [file pone.0191107.s003.docx]

Supporting information Fig S3


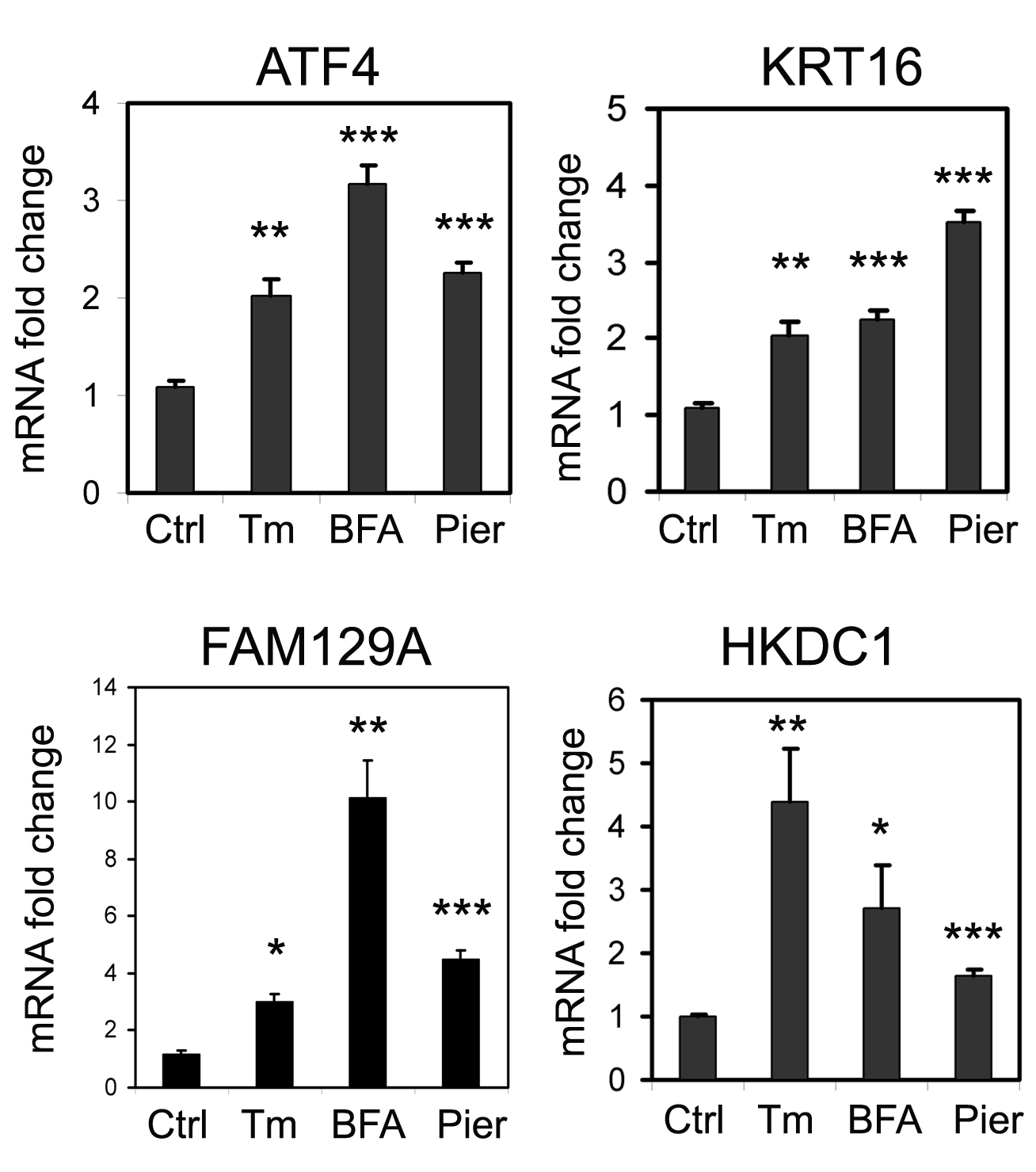


**Fig S3. Induction of ATF4, KRT16, FAM129A and HKDC1 transcripts by ER stress or inhibition of mitochondrial respiratory chain in HeLa cells.** Fold changes of ATF4, KRT16, FAM129A and HKDC1 transcripts in HeLa cells treated with Tunicamycin (Tm) for 14h, Brefeldin A (BFA) or Piericidin A (Pier) for 8h. The data was obtained by RT-qPCR and processed as described in Materials and Methods.
