# Supplementary material for: Implication of KRT16, FAM129A and HKDC1 genes as ATF4 regulated components of the integrated stress response
Source: PLoS One. 2018 Feb 8;13(2):e0191107. doi: 10.1371/journal.pone.0191107 (PMC5805170; doi:10.1371/journal.pone.0191107)
Supplement: S4 Fig — Fold changes of ATF4, KRT16, FAM129A and HKDC1 transcripts in HEK293T cells treated with Tunicamycin (Tm), Brefeldin A (BFA) or Piericidin A (Pier) for 14 h. The data was obtained by RT-qPCR and processed as described in Materials and Methods. (DOCX) [file pone.0191107.s004.docx]

Supporting information Fig S4

**
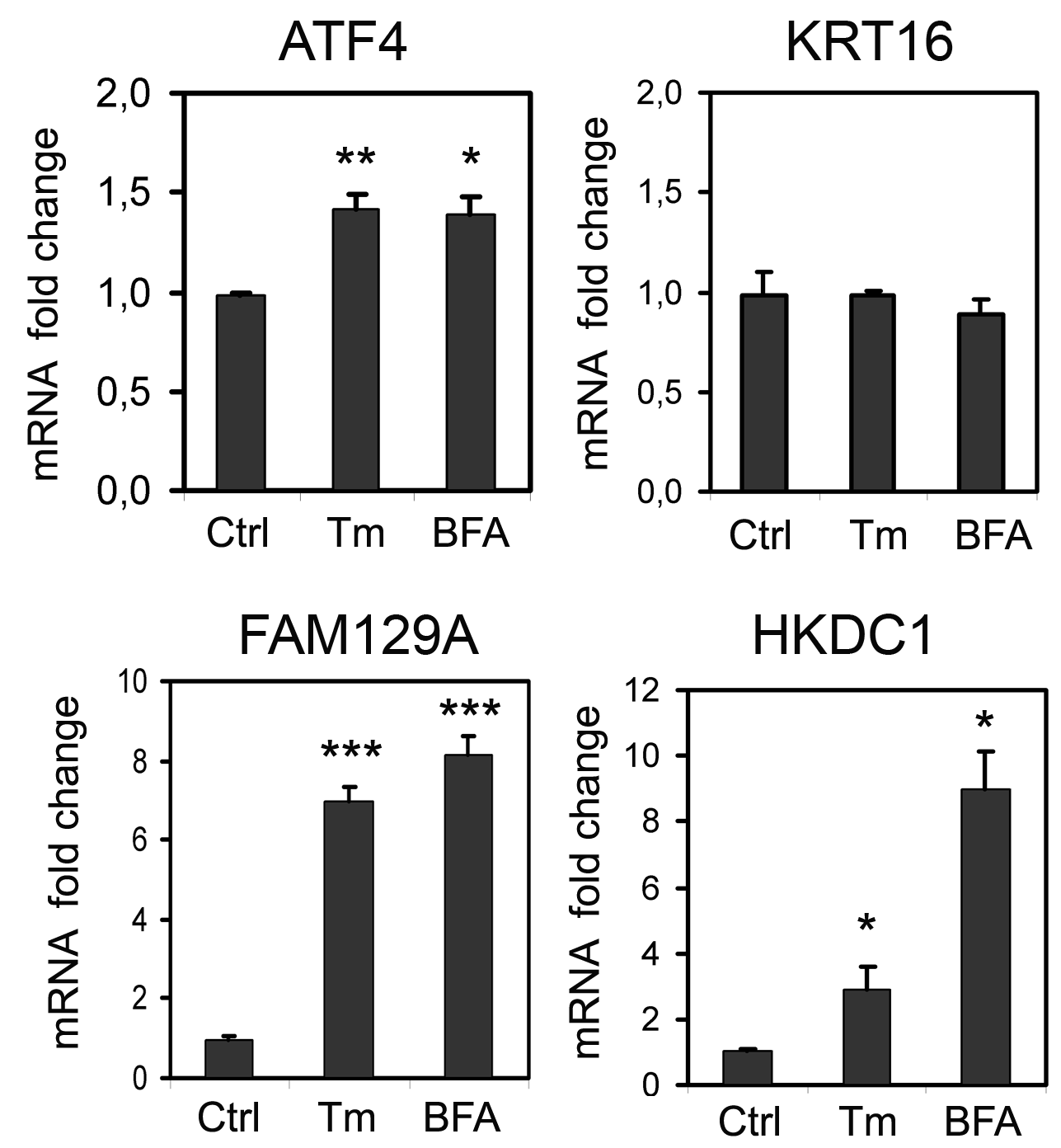
**

**Fig S4. Induction of ATF4, KRT16, FAM129A and HKDC1 transcripts by ER stress or inhibition of mitochondrial respiratory chain in HEK293T cells.** Fold changes of ATF4, KRT16, FAM129A and HKDC1 transcripts in HEK293T cells treated with Tunicamycin (Tm), Brefeldin A (BFA) or Piericidin A (Pier) for 14 h. The data was obtained by RT-qPCR and processed as described in Materials and Methods.
