# Supplementary material for: Implication of KRT16, FAM129A and HKDC1 genes as ATF4 regulated components of the integrated stress response
Source: PLoS One. 2018 Feb 8;13(2):e0191107. doi: 10.1371/journal.pone.0191107 (PMC5805170; doi:10.1371/journal.pone.0191107)
Supplement: S5 Fig — Fold changes of KRT6A transcripts in HCT116 cells treated with Tunicamycin (Tm) or Brefeldin A (BFA) for 14 h. The data was obtained by RT-qPCR and processed as described in Materials and Methods. (DOCX) [file pone.0191107.s005.docx]

Supporting information Fig S5

**
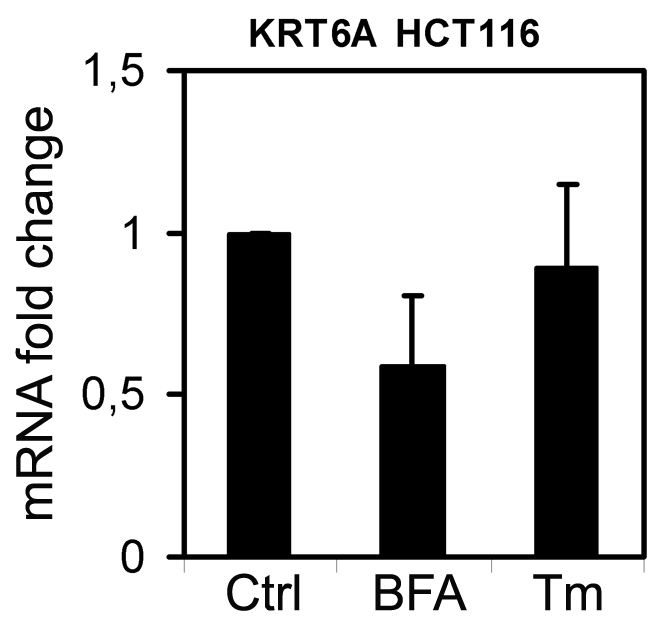
**

**Fig S5. Levels of KRT6A mRNA in HCT116 cells treated with ER stress inducers.** Fold changes of KRT6A transcripts in HCT116 cells treated with Tunicamycin (Tm) or Brefeldin A (BFA) for 14 h. The data was obtained by RT-qPCR and processed as described in Materials and Methods.
